# A Multidisciplinary Healthy Aging Program in Comprehensive HIV Care: Multidomain Screening, Clinical Interventions, and Cardiometabolic Risk Management

**DOI:** 10.3390/v18050572

**Published:** 2026-05-19

**Authors:** Steven Y. Hong, Deborah Woodley, Megan Pao, Holly Goetz, Alejandro Alvarez, Max White, Bruce Hirsch, Edith Burns, Joseph P. McGowan

**Affiliations:** 1 Karin and Dayton Brown, Jr. Division of Infectious Diseases, Northwell Health, New Hyde Park, NY 11030, USA; dwoodley@northwell.edu (D.W.); mlam2@northwell.edu (M.P.); hgoetz@northwell.edu (H.G.); aalvarez13@northwell.edu (A.A.); mwhite23@northwell.edu (M.W.); bhirsch@northwell.edu (B.H.); eburns4@northwell.edu (E.B.); jmcgowan@northwell.edu (J.P.M.); 2 Northwell Health HIV Service Line Program, Center for AIDS Research and Treatment (CART), Northwell Health, New Hyde Park, NY 11030, USA; 3 Ambulatory Pharmacy Services, VIVOHealth Pharmacy, Northwell Health, Lake Success, NY 11042, USA

**Keywords:** HIV, aging, people living with HIV, geriatric screening, multidomain screening, healthy aging

## Abstract

Background: People living with HIV (PLWH) are increasingly reaching older ages due to the success of antiretroviral therapy. However, aging with HIV is associated with increased risk of multimorbidity, neurocognitive impairment, frailty, psychosocial stress, and functional decline. Multidomain geriatric screening framed within an Age-Friendly 4Ms Framework (Mentation, Medication, Mobility, What Matters Most) and consideration of multi-complexity may help identify aging-related vulnerabilities and guide multidisciplinary care with greater impact on patient outcomes. However, real-world implementation of such programs within HIV clinical settings remains limited. Methods: We conducted a retrospective analysis of adults aged ≥50 years enrolled in a multidisciplinary Healthy Aging Program within a large, integrated HIV care system. Multidomain screening assessments included cognitive evaluation (Montreal Cognitive Assessment), mental health screening (PHQ-2, GAD-2), functional assessment (Katz ADL, Lawton IADL), frailty screening (Edmonton Frail Scale), and intrinsic capacity domains using the WHO Integrated Care for Older People (ICOPE) framework. Screening results, referrals, clinical interventions, and cardiometabolic risk management measures were extracted from clinical program databases and electronic medical records. Results: A total of 317 adults aged ≥50 years completed multidomain screening. Participants had well-controlled HIV infection, with viral suppression in 96.2% and a median CD4 count of 660 cells/mm^3^. Despite this, aging-related vulnerabilities were common. Overall, 78.4% of participants had at least one abnormal screening domain. Cognitive impairment was identified in nearly half of individuals screened, including mild impairment in 39.8% and moderate impairment in 8.7%. Functional limitations were identified in 10.1% of participants, while anxiety symptoms were present in 9.5%. Sensory impairments were common, including vision impairment in 36.5% of participants. Polypharmacy was prevalent, with 33.2% of participants prescribed five or more chronic medications. Screening frequently generated multidisciplinary referrals, including behavioral health services (42.3%), social work support (42.9%), and pharmacist-led cardiometabolic risk review (56.8%). Age-stratified analyses demonstrated similar prevalence of screening abnormalities across age groups, including individuals aged 50–59 years. Modest improvements in cardiometabolic preventive care were observed during follow-up. Statin utilization increased from 65.6% at baseline to 70.0% at 12 months, and LDL cholesterol declined modestly during the observation period. Conclusions: Multidomain screening integrated into routine HIV care identified a high prevalence of aging-related vulnerabilities among PLWH aged ≥50 years despite excellent virologic control. These findings suggest that aging-related risk in HIV is not adequately captured by chronological age alone and support early, universal implementation of multidomain screening within HIV care models.

## 1. Introduction

Advances in antiretroviral therapy (ART) have transformed HIV infection into a chronic manageable disease, resulting in dramatic increases in life expectancy for people living with HIV (PLWH). As a result, the demographic profile of the HIV epidemic has shifted substantially, with a growing proportion of PLWH now reaching older age. In the United States and other high-income settings, more than half of individuals receiving HIV care are now aged 50 years or older. Projections suggest that this trend will continue, with substantial increases in the proportion and absolute number of older PLWH over the coming decades, including marked growth in those aged ≥65 and ≥75 years [1]. This shift has led to increasing recognition that HIV care must address not only virologic control but also the complex medical, functional, and psychosocial needs associated with aging with HIV.

A growing body of evidence demonstrates that PLWH experience a higher burden of age-associated comorbidities compared with the general population. These include cardiovascular disease, metabolic disorders, neurocognitive impairment, frailty, and functional limitations [2,3,4]. Several mechanisms have been proposed to contribute to this increased burden, including persistent immune activation, chronic inflammation, long-term ART exposure, and higher prevalence of traditional risk factors [5]. As a result, PLWH may develop multimorbidity and geriatric syndromes at younger ages than HIV-negative individuals.

Neurocognitive impairment remains particularly prevalent among aging individuals with HIV despite virologic suppression on ART [6,7]. Similarly, frailty, functional decline, and polypharmacy are increasingly recognized as important clinical challenges in this population [8,9]. Prior cohort studies have demonstrated that multimorbidity and geriatric conditions are common among older adults living with HIV and may emerge earlier in life compared with the general population [2,3].

In response to these challenges, there has been increasing interest in integrating geriatric principles into HIV care. The 4Ms Framework of the Institute for Healthcare Improvement’s Age-Friendly Health Systems Initiative offers an evidence-based approach for assessing the needs of complex aging populations [10]. Geriatric assessment of Mentation, Mobility, Medication and What Matters Most, along with social determinants of health and factors contributing to multi-complexity provide opportunities to optimize care for PLWH. The World Health Organization Integrated Care for Older People (ICOPE) framework represents an approach designed to identify multi-complexity by assessing declines in intrinsic capacity across multiple domains and suggesting targeted interventions [11]. However, there remains limited real-world evidence describing the implementation of such structured multidomain screening within routine HIV care settings. Despite growing recognition of the need for integrated aging-focused HIV care, relatively few studies have described the implementation of structured multidomain screening programs within large real-world HIV clinical systems.

The Healthy Aging Program (HAP) for PLWH was developed within the infectious diseases division of a large, integrated health system to address the evolving needs of older adults living with HIV through a multidisciplinary care model integrating geriatric screening, pharmacy review, behavioral health services, social work support, and cardiometabolic risk management. The program is embedded within a large, integrated HIV care network in the New York metropolitan region, providing care to several thousand PLWH.

In this study, we describe the implementation of structured multidomain aging screening within this clinical program. Specifically, we sought to describe the prevalence of aging-related vulnerabilities identified through screening, characterize referrals and multidisciplinary services offered to clients, and assess early changes in cardiometabolic risk factors and preventive therapy utilization among program participants. We also examined whether the prevalence of screening abnormalities differed across age strata and by duration of HIV infection to better inform optimal timing and targeting of aging-related screening in PLWH.

## 2. Methods

### 2.1. Study Design and Setting

This study is a retrospective observational analysis of data collected through a multidisciplinary Healthy Aging Program (HAP), implemented within a large, integrated HIV care program at an academic medical center. The HIV care program is based within a large academic HIV clinic in the New York metropolitan region and provides comprehensive multidisciplinary care for several thousand PLWH. Screening and clinical data collected between July 2023 and March 2026 were included in the present analysis.

Participants aged 50 years and older receiving care within the HIV care program were invited to participate in the HAP as part of routine clinical care. The HAP was implemented gradually across the clinical site, and participant identification and enrollment workflows evolved during early program implementation as the HAP was integrated into routine clinical practice. The program was developed to address the growing needs of individuals aging with HIV by integrating geriatric-oriented screening and multidisciplinary care coordination into HIV clinical practice.

### 2.2. Multidisciplinary Care Model

**Figure 1:**
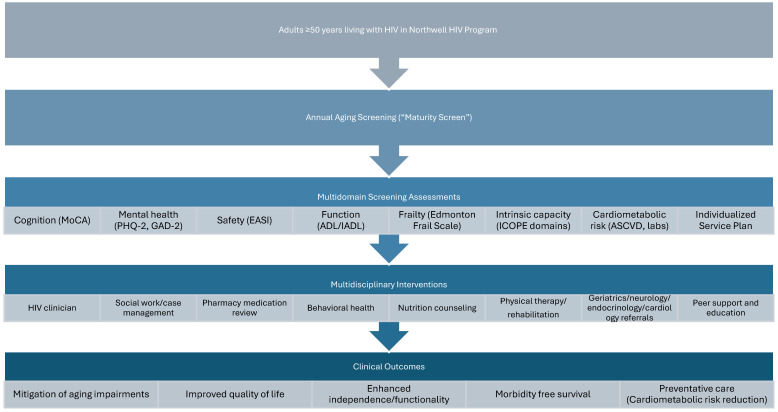
Multidomain Screening and Care Pathway in the HIV Healthy Aging Program. Multidomain screening includes cognitive (MoCA), mental health (PHQ-2, GAD-2), functional status (Katz ADL, Lawton–Brody IADL), frailty (Edmonton Frail Scale), social vulnerability (EASI), and intrinsic capacity domains (WHO ICOPE: mobility, cognition, sensory function, nutrition, mood). Screening results inform multidisciplinary referrals, including behavioral health, social work, pharmacy-led cardiometabolic risk management, rehabilitation services, and subspecialty care. Abbreviations: MoCA, Montreal Cognitive Assessment; PHQ-2, Patient Health Questionnaire-2; GAD-2, Generalized Anxiety Disorder-2; ADL, activities of daily living; IADL, instrumental activities of daily living; EASI, Elder Abuse Suspicion Index; ICOPE, Integrated Care for Older People.

The HAP was designed as a multidisciplinary care model integrating geriatric screening with coordinated clinical interventions (Figure 1). Screening assessments were conducted as part of routine clinical care and reviewed by a multidisciplinary team that included HIV physicians, geriatricians, clinical pharmacists, social workers, nursing staff, and program coordinators.

Following completion of the multidomain screening assessment, results were reviewed to identify potential cognitive, functional, psychological, or social vulnerabilities. Screening results were reviewed by the multidisciplinary HAP team, and referrals were guided by screening findings, clinical judgment, patient priorities, and existing specialty care involvement. Potential interventions included medication review and optimization by clinical pharmacists, social work evaluation for psychosocial needs and community support resources, behavioral health referrals for anxiety or mood disorders, physical and occupational therapy referrals for mobility or functional limitations, and coordination with primary care or specialty services for management of cardiometabolic conditions.

Clinical pharmacists embedded within the HIV clinics played a central role in preventive care initiatives and medication management. Pharmacists reviewed medication lists for polypharmacy, assessed potential drug–drug interactions, and collaborated with providers to initiate or optimize therapies for chronic conditions, including statin therapy for cardiovascular risk reduction and GLP-1 receptor agonists for metabolic disease when appropriate. Pharmacists practiced under collaborative drug therapy management agreements that allowed them to assist in medication optimization and laboratory monitoring in coordination with supervising physicians. Social workers also played an important role in addressing psychosocial determinants of health and fostering community engagement among program participants. In addition to providing individualized support services, social workers facilitated group-based activities and educational sessions designed to promote healthy aging, including discussions on nutrition, physical activity, and social connectedness.

Through this multidisciplinary structure, the HAP aimed to move beyond traditional HIV care focused primarily on virologic control and instead provide a more comprehensive approach addressing the complex medical, functional, and psychosocial needs of aging PLWH.

### 2.3. Screening Assessments

Participants enrolled in the HAP completed a multidomain screening assessment designed to evaluate cognitive, functional, psychological, and social domains relevant to aging with HIV. Screening tools were selected based on their established use in geriatric and HIV populations and were administered by trained program staff. Completion of individual tools varied based on phased rollout, staffing, workflow, language considerations, and patient willingness.

Cognitive function was assessed using a modified Montreal Cognitive Assessment (MoCA), a widely used screening instrument for mild cognitive impairment that evaluates multiple cognitive domains including memory, executive function, attention, language, and visuospatial abilities [12]. The MoCA has been used extensively in studies of HIV-associated neurocognitive impairment [6]. Depression symptoms were assessed using the Patient Health Questionnaire-2 (PHQ-2), a brief validated screening tool used to identify individuals at risk for depressive disorders [13]. Anxiety symptoms were assessed using the Generalized Anxiety Disorder 2-item scale (GAD-2), a brief validated screening tool used in clinical settings to identify patients with potential anxiety disorders [14]. Risk of elder abuse was evaluated using the Elder Abuse Suspicion Index (EASI), a validated instrument designed to identify potential mistreatment in older adults during routine clinical encounters [15]. Functional status was assessed using both the Katz Index of Activities of Daily Living (ADL) and the Lawton–Brody Instrumental Activities of Daily Living (IADL) scale [16,17]. The Katz Index evaluates independence in basic self-care activities such as bathing, dressing, and feeding, while the Lawton–Brody scale assesses higher-level functional abilities, including medication management, transportation, and financial management. Frailty and overall vulnerability were evaluated using the Edmonton Frail Scale, a multidimensional screening instrument that assesses cognition, general health status, functional independence, medication use, nutrition, mood, and mobility [18]. Participants also completed screening using a modified Integrated Care for Older People (ICOPE) framework, developed by the World Health Organization to identify declines in intrinsic capacity across domains including cognition, mobility, sensory function, psychological well-being, and vitality [11].

In addition to these screening instruments, demographic characteristics, medical comorbidities, medication use, and laboratory data were extracted from the electronic medical record. Cardiometabolic risk factors including hypertension, diabetes, lipid levels, and ASCVD risk scores were also captured. Pharmacy surveys were used to assess medication burden, polypharmacy, and use of preventive therapies such as statins and GLP-1 receptor agonists.

### 2.4. Data Sources and Outcomes

Clinical and screening data were obtained from the program’s REDCap database and the Allscripts electronic medical record. Screening assessments and subsequent interventions were recorded in REDCap, while demographic information, comorbidities, laboratory values, and medication data were extracted from the electronic medical record. Variables collected included age, sex, HIV disease markers, comorbid conditions, medication lists, cardiometabolic risk factors, and preventive therapy utilization. Medication count data were available only for participants completing structured HAP/pharmacy assessment, as some participants underwent standalone ICOPE screening during early program implementation when staffing and workflow limitations precluded full multidisciplinary assessment. Cardiovascular risk was estimated using the ASCVD risk calculator when applicable.

Descriptive statistics were used to summarize demographic and clinical characteristics, screening results, and interventions. Analyses were performed using SAS version 3.8 (Enterprise Edition. Copyright 2012–2018, SAS Institute Inc., Cary, NC, USA).

### 2.5. Ethical Review

This study was reviewed by the Northwell Health Institutional Review Board and was granted exempt status, as it involved retrospective analysis of de-identified data collected as part of routine clinical care and program evaluation.

## 3. Results

### 3.1. Participant Characteristics

A total of 317 adults aged ≥50 years completed multidomain screening through the HAP during the study period. Baseline characteristics of the cohort are summarized in Table 1. Not all screening assessments were completed for all participants due to phased implementation of certain tools and variation in clinical workflows during the program’s early rollout of the HAP. As a result, denominators for specific screening domains vary across analyses.

The median age was 63 years (IQR 58–68), with participants distributed across three age groups: 50–59 years (33.8%), 60–64 years (23.3%), and ≥65 years (42.9%). The cohort was predominantly male (56.5%), with nearly half of participants identifying as non-Hispanic Black (48.9%), followed by non-Hispanic White (32.5%) and Hispanic (13.3%). Viral suppression was achieved in 96.2% of participants, and the median CD4 count at the time of screening was 660 cells/mm^3^ (IQR 461–865). Participants had long-standing HIV infection, with a median duration since diagnosis of 37.3 years (IQR 24.7–49.6). Hypertension was present in 59.9% of participants, hyperlipidemia in 65.0%, and diabetes mellitus in 21.5%. The median estimated 10-year ASCVD risk score at enrollment was 11.75% (IQR 6.06–19.20), indicating moderate cardiovascular risk across the cohort. One-third of participants (33.2%) were prescribed five or more chronic medications, consistent with polypharmacy, while an additional 11.5% were prescribed three to four medications. Only 2.4% of participants reported taking no chronic medications. Overweight and obesity were common, with 38.8% of participants classified as overweight (BMI 25–29.9 kg/m^2^) and 35.6% classified as obese (BMI ≥ 30 kg/m^2^).

### 3.2. Prevalence of Aging-Related Screening Abnormalities

Multidomain screening identified aging-related vulnerabilities across cognitive, functional, psychosocial, and sensory domains (Table 2). Cognitive screening using the Montreal Cognitive Assessment was completed in 103 participants. While approximately half of individuals screened had normal cognitive scores (51.5%), mild cognitive impairment was identified in 39.8% of screened participants. Moderate impairment was identified in an additional 8.7% of individuals.

Depression screening using the PHQ-2 was positive in 4.6% of participants, while anxiety symptoms based on the GAD-2 were identified in 9.5%. Screening for potential social vulnerability using the Elder Abuse Suspicion Index identified positive responses among 5.3% of participants. Functional limitations were also identified through screening assessments. Dependence on at least one basic activity of daily living was observed in 10.1% of participants, while instrumental functional limitations were identified in 4.7%. Frailty screening using the Edmonton Frail Scale demonstrated that most individuals were classified as not frail (92.5%). However, 5.4% were categorized as vulnerable, with smaller proportions demonstrating mild (1.6%) or moderate (0.5%) frailty. ICOPE-based screening identified additional vulnerabilities across mobility, sensory, and nutritional domains. Mobility impairment was identified in 17.7% of participants, nutritional risk in 18.7%, mood concerns in 13.9%, and memory concerns in 11.2%. Sensory impairments were particularly common, with vision impairment reported in 36.5% and hearing impairment in 12.7%. Overall, 78.4% of participants had at least one abnormal screening domain, highlighting the high burden of multidomain impairment in this population.

### 3.3. Age-Stratified Screening Outcomes

Age-stratified screening outcomes are presented in Table 3. Across most domains, the prevalence of screening abnormalities was broadly similar across age groups. For example, mild cognitive impairment based on MoCA screening was observed in 41.7% of individuals aged 50–59 years, 43.5% of those aged 60–64 years, and 36.4% of those aged ≥65 years. Moderate impairment occurred in 2.8%, 8.7%, and 13.6% of individuals in these respective age groups. These differences were not statistically significant (*p* = 0.54). Similarly, anxiety symptoms were observed in 10.3% of individuals aged 50–59 years, 4.7% of those aged 60–64 years, and 11.2% of those aged ≥65 years (*p* = 0.46). Functional limitations based on ADL and IADL assessments were present in 10.5%, 16.3%, and 6.7% of individuals across the three age groups (*p* = 0.23). Frailty classification also did not differ significantly by age group. The proportion of participants classified as not frail ranged from 89.7% to 97.7% across age strata (*p* = 0.62). Similarly, the proportion of individuals with at least one abnormal ICOPE domain was comparable across age groups, affecting 77.9% of individuals aged 50–59 years, 81.8% of those aged 60–64 years, and 76.8% of those aged ≥65 years (*p* = 0.79). Depression and safety screening outcomes also did not differ significantly across age groups, with low overall prevalence of PHQ-2 positivity and EASI-positive responses observed across all strata.

We further examined whether the burden of aging-related impairments differed by duration of HIV infection, stratified as <10 years, 10–19 years, and ≥20 years since diagnosis (Table 4). Overall, there were no statistically significant differences in the prevalence of impairment across domains between groups. Cognitive performance was similar, with comparable proportions of participants classified as normal, mildly impaired, and moderately impaired by MoCA. Likewise, the prevalence of depressive symptoms, anxiety, frailty, functional limitations (ADL and IADL), and multidomain ICOPE did not differ significantly by duration of HIV infection. These findings indicate that the burden of aging-related impairments in this cohort was not significantly different by years since HIV diagnosis.

### 3.4. Referrals, Services, and Clinical Interventions

Screening findings frequently were followed by multidisciplinary interventions and referrals (Table 5). Mental health services represented one of the most common referral pathways, with 42.3% of participants referred to psychiatry or behavioral health services. Similarly, 42.9% of participants received social work support addressing social needs or safety concerns. Clinical pharmacist review for cardiometabolic risk management was conducted for more than half of participants (56.8%), reflecting the integration of pharmacy services into the Healthy Aging Program model. Additional referrals included nutrition counseling (12.6%), physical or occupational therapy for functional limitations (8.8%), and exercise or rehabilitation programs (1.9%). Other supportive services included food insecurity support (2.5%) and peer support programs (1.0%). Medical subspecialty referrals were also common, occurring in 30.3% of participants, reflecting the complexity of multimorbidity in this population. Clinical care coordination was provided for approximately half of participants (50.2%), demonstrating the integration of screening results into ongoing clinical management.

### 3.5. Cardiometabolic Parameters and Preventive Therapy

Cardiometabolic parameters at baseline and follow-up are summarized in Table 6. Among participants with available data, modest improvements in several cardiometabolic risk markers were observed during follow-up. Median LDL cholesterol decreased from 98 mg/dL (IQR 75–123) at baseline to 93.5 mg/dL at six months and 90.5 mg/dL at twelve months, corresponding to a median reduction of approximately 7 mg/dL over one year. Systolic and diastolic blood pressure remained largely stable over time. Median systolic blood pressure decreased slightly from 133 mmHg at baseline to 132 mmHg at six months, while diastolic blood pressure remained approximately 80 mmHg throughout follow-up. Body mass index demonstrated modest reductions over time, decreasing from a median of 27.9 kg/m^2^ at baseline to 27.3 kg/m^2^ at twelve months. Glycemic control remained stable, with median hemoglobin A1c values of approximately 5.7% across time points. Median ASCVD risk scores remained relatively stable over time, with minimal change observed during follow-up.

### 3.6. Preventive Cardiometabolic Therapy Utilization

**Figure 2:**
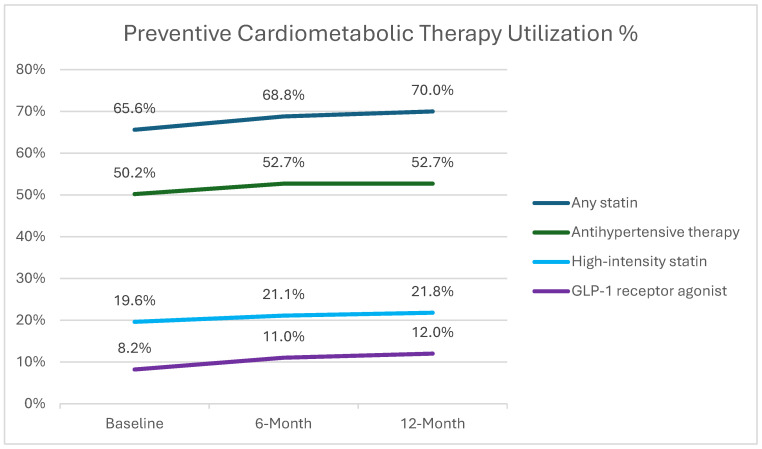
Preventive Cardiometabolic Therapy Utilization. Preventive cardiometabolic therapies include statins (overall and high-intensity), glucagon-like peptide-1 (GLP-1) receptor agonists, and antihypertensive medications assessed at baseline and during follow-up. Proportions reflect the percentage of participants prescribed each therapy at each time point among those with available data. Abbreviations: GLP-1, glucagon-like peptide-1 receptor agonist.

Preventive cardiometabolic therapy utilization increased modestly during follow-up (Figure 2). At baseline, 65.6% of participants were prescribed statin therapy. This proportion increased to 68.8% at six months and 70.0% at twelve months. Use of high-intensity statins also increased slightly, rising from 19.6% of participants at baseline to 21.8% at twelve months. Similarly, use of GLP-1 receptor agonists increased from 8.2% at baseline to 12.0% at twelve months. Antihypertensive therapy utilization remained largely stable over time, increasing only slightly from 50.2% at baseline to 52.7% during follow-up.

## 4. Discussion

In this descriptive analysis of a multidisciplinary HAP embedded within a large HIV care network, we found that structured multidomain screening identified a high burden of aging-related vulnerabilities among adults aged 50 years and older living with HIV. Despite excellent HIV control in this cohort, with viral suppression achieved in more than 96% of participants and a median CD4 count of 660 cells/mm^3^, screening revealed substantial prevalence of cognitive, functional, psychosocial, and sensory impairments. Nearly four out of five participants (78%) had at least one abnormal screening domain, underscoring the substantial burden of aging-related vulnerabilities among PLWH engaged in routine outpatient care and highlighting the importance of systematic geriatric assessment in this population. Importantly, these findings suggest that virologic control alone does not fully capture the burden of aging-related vulnerability in PLWH.

These findings are consistent with a growing body of literature demonstrating that PLWH experience an increased burden of age-associated comorbidities and geriatric syndromes compared with HIV-negative populations [2,3,4]. Even in the modern ART era, persistent immune activation, chronic inflammation, and multimorbidity contribute to the development of functional impairments and geriatric vulnerabilities [5]. Our findings extend prior work by demonstrating that structured multidomain screening integrated into routine HIV care can identify clinically relevant vulnerabilities across multiple domains, even among individuals with well-controlled HIV infection.

Cognitive impairment was particularly common in our cohort. Among participants undergoing MoCA screening, nearly half demonstrated mild or moderate cognitive impairment. This prevalence is consistent with prior studies documenting persistent neurocognitive impairment among aging individuals with HIV despite effective ART [6,7], and appears higher than estimates reported among community-dwelling adults aged ≥50 years in the general population [19]. These findings highlight the continued relevance of routine cognitive screening in HIV care settings, particularly as the population of people aging with HIV continues to grow.

Functional and psychosocial vulnerabilities were also frequently identified. Dependence in activities of daily living, anxiety symptoms, social vulnerability, and sensory impairments were all detected through screening assessments. Importantly, these screening findings frequently resulted in multidisciplinary referrals and services, including behavioral health support, social work services, physical therapy, nutrition counseling, and pharmacy review. More than half of participants underwent pharmacist-led cardiometabolic risk review, and substantial proportions were referred to behavioral health or social services. Medical subspecialty referrals were also common, occurring in approximately one-third of participants, further reflecting the high burden of multimorbidity and complexity of care needs in this population. Referral frequencies did not necessarily correspond directly to individual screening instrument positivity, as referrals often reflected broader psychosocial or clinical assessment beyond isolated screening results. Formal exercise referrals were relatively uncommon and likely underestimate broader exercise and mobility counseling provided during routine care. These findings demonstrate the feasibility of integrating multidomain geriatric screening into routine HIV care within a multidisciplinary care model. Frailty prevalence may also vary by instrument, as the Edmonton Frail Scale differs from phenotype-based frailty definitions commonly used in HIV aging cohorts.

An important finding of this study was that aging-related vulnerabilities were observed across all age strata, including among individuals aged 50–59 years. Age-stratified analyses demonstrated similar prevalence of screening abnormalities across age groups, including cognitive impairment, functional limitations, frailty, and ICOPE-based vulnerabilities. These findings support recommendations that aging-focused screening in PLWH begin earlier than in the general population, often at age 50 rather than age 65 [8,9]. The absence of substantial differences across age groups in our cohort suggests that clinically meaningful vulnerabilities are already present among individuals in their fifties living with HIV and that delaying screening until later ages may miss opportunities for early identification and intervention.

Notably, we did not observe meaningful differences in the prevalence of aging-related impairments when stratified by duration of HIV infection (<10 years, 10–19 years, and ≥20 years). This finding suggests that years since diagnosis alone may not adequately capture the risk of age-associated vulnerability in people with HIV. Rather, the burden of impairment observed in this cohort likely reflects a multifactorial process involving early immune injury, chronic inflammation, multimorbidity, and social determinants of health. We did not observe a clear gradient by duration of HIV infection, which further underscores the limitations of duration-based risk stratification and supports the use of universal, multidomain screening approaches for all older adults with HIV, regardless of time since diagnosis. Together with the age-stratified findings, these results suggest that broad categories of age and HIV duration alone may not adequately differentiate risk.

Polypharmacy was also highly prevalent in this cohort. Approximately one-third of participants were prescribed five or more chronic medications, reflecting the substantial burden of multimorbidity among aging PLWH. Prior studies have similarly demonstrated high rates of polypharmacy in this population, driven by both HIV-related comorbidities and age-associated chronic conditions [2,3]. Medication review and deprescribing strategies therefore represent an important component of multidisciplinary HIV aging care.

We also observed modest improvements in cardiometabolic risk management over time. Statin utilization increased from approximately 66% at baseline to 70% at 12 months, while use of GLP-1 receptor agonists increased from 8% to nearly 12%. In parallel, LDL cholesterol levels declined modestly over the observation period. In contrast, blood pressure, glycemic control, and estimated ASCVD risk remained largely stable over time, suggesting that short-term changes in global cardiometabolic risk may be limited despite increased preventive therapy utilization. These improvements likely reflect targeted cardiometabolic risk management facilitated by the multidisciplinary care model, including clinical pharmacist involvement. However, the magnitude of change was modest, which may be attributable in part to the relatively short follow-up period available for analysis. The high prevalence of overweight and obesity in this cohort further underscores the importance of integrated cardiometabolic risk management, including the observed increase in GLP-1 receptor agonist use over time. Subgroup analyses among participants with diabetes or hypertension were not included in the current retrospective analysis, and sodium-glucose cotransporter-2 (SGLT2) inhibitor use and indication-specific prescribing were not systematically captured.

Our findings also highlight the potential role of structured screening frameworks such as ICOPE in HIV care settings. Originally developed by the World Health Organization to guide integrated care for older adults, the ICOPE framework provides a structured approach to identifying declines in intrinsic capacity across domains including mobility, cognition, sensory function, and nutrition [11]. Our experience suggests that such frameworks can be feasibly implemented within HIV clinics and may help standardize multidomain assessment for aging populations with HIV.

This study has several limitations. First, the analysis was based on retrospective review of clinical screening data collected as part of routine program implementation, and not all screening tools were completed for all participants. The HAP was implemented gradually as a new clinical program with evolving enrollment workflows, which limited the availability of complete denominator data for eligible or approached participants and may have preferentially included individuals with greater medical or psychosocial needs. Additionally, while referrals and clinical interventions were temporally associated with screening results, direct linkage between specific screening abnormalities and subsequent referrals could not be definitively established, and these relationships are inferred based on timing within clinical workflows. Vision- and hearing-specific referrals were not consistently tracked separately within the retrospective database. Furthermore, the ICOPE assessment was implemented using a modified version adapted for clinical workflow, which may limit comparability to standard ICOPE-based assessments and reduce generalizability of these findings. MoCA performance may also be influenced by education, language, and cultural factors, and assessments were primarily conducted in English. Second, follow-up duration for cardiometabolic outcomes was relatively limited, which may have constrained the magnitude of observable changes in preventive therapy and clinical parameters. The study sample size, while reflective of a real-world clinical program, may limit power for certain subgroup and longitudinal analyses. Third, this study reflects the experience of a single large health system, which may limit generalizability to smaller clinical programs. The absence of a comparator group of patients not undergoing structured screening limits the ability to attribute observed changes directly to the program, and no matched control group without HIV was included to contextualize the burden of impairments relative to the general aging population. However, the scale and diversity of the HAP, which serves a large population of PLWH within a large integrated health system, supports the broader relevance of these findings.

Despite these limitations, our study demonstrates that multidomain geriatric screening can be successfully integrated into routine HIV care within a large health system and can identify a substantial burden of aging-related vulnerabilities among PLWH aged 50 years and older. These findings highlight the feasibility of implementing structured multidomain aging screening within HIV clinical practice and support the integration of multidisciplinary services addressing functional health, mental health, social needs, and cardiometabolic risk among aging individuals with HIV. The high prevalence of multidomain impairment observed in this cohort further emphasizes the need for proactive, rather than reactive, approaches to aging-related care in PLWH.

Future research should evaluate the long-term impact of multidomain screening programs on clinical outcomes among people aging with HIV. Longer follow-up will be needed to determine whether improvements in preventive cardiometabolic therapy translate into reductions in cardiovascular events, functional decline, hospitalization, and quality-of-life outcomes. In addition, further studies may help refine optimal screening strategies and determine how multidisciplinary aging programs can be implemented and scaled across diverse HIV care settings. Future program development should also incorporate more systematic fall risk and fracture prevention assessment. Our experience also highlighted the operational challenges of implementing comprehensive multidomain screening within routine HIV care. Moving forward, the program plans to further develop a staged screening approach to balance resource requirements with the goal of broadly screening all PLWH aged 50 years and older.

As the global population of individuals aging with HIV continues to expand, care models must evolve beyond virologic management to address multimorbidity, functional health, and social determinants of health. Multidisciplinary programs integrating structured multidomain screening may provide scalable and effective approaches to delivering comprehensive, patient-centered care for aging PLWH.

## Figures and Tables

**Table 1:** Baseline Characteristics of Adults Aged ≥ 50 Years Screened.

Characteristic	Overall (*N*=)
Age, median (IQR)	63 (58–68)
Age group, n (%)	
50–59 years	107 (33.8)
60–64 years	74 (23.3)
≥65 years	136 (42.9)
Sex at birth, n (%)	
Male	179 (56.5)
Female	138 (43.5)
Race/ethnicity, n (%)	
Non-Hispanic White	103 (32.5)
Non-Hispanic Black	155 (48.9)
Hispanic	42 (13.3)
Other	11 (3.5)
Unknown	6 (1.9)
Years since HIV diagnosis, median (IQR)	24.2 (16.0–32.1)
HIV viral load suppressed, n (%)	303 (96.2)
CD4 count (time of screening), median (IQR)	660 (461–865)
Hypertension, n (%)	190 (59.9)
Diabetes mellitus, n (%)	68 (21.5)
Hyperlipidemia, n (%)	206 (65)
ASCVD risk score %, median (IQR)	11.75 (6.1–19.2)
Number of chronic medications, n (%)	
None	9 (2.4)
1–2	25 (6.7)
3–4	43 (11.5)
5+	124 (33.2)
Unknown	173 (46.3)
BMI Categories, n (%)	
<18.5	11 (2.9)
18.5–24.9	85 (22.7)
25–29.9	145 (38.8)
30–39.9	115 (30.8)
40+	18 (4.8)

Data are presented as median (interquartile range [IQR]) or n (%). Abbreviations: ASCVD, atherosclerotic cardiovascular disease; BMI, body mass index; CD4, CD4+ T-lymphocyte count. Medication count was unavailable for some participants who underwent standalone ICOPE screening without full HAP/pharmacy assessment.

**Table 2:** Baseline Prevalence of Aging-Related Impairments by Screening Domain.

Screening Domain	Tool		Abnormal Definition	N Abnormal (%)
Cognition	MoCA	103	≥26 (normal)	53 (51.5)
			18–25 (mild)	41 (39.8)
			10–17 (moderate)	9 (8.7)
			<10 (severe)	0
Depression	PHQ-2	152		7 (4.6)
Anxiety	GAD-2	190	≥3	18 (9.5)
Safety/Social risk	EASI	190	≥1 positive	10 (5.3)
Basic function	Katz ADL	189	Any dependence	19 (10.1)
Instrumental function	Lawton IADL	190	Any dependence	9 (4.7)
Frailty	Edmonton Frail Scale	187	0–5 not frail	173 (92.5)
			6–7 vulnerable	10 (5.4)
			8–9 mild frailty	3 (1.6)
			10–11 moderate frailty	1 (0.5)
			12–17 severe	0
Mobility	ICOPE	306	Abnormal	64 (17.7)
Nutrition	ICOPE	311	Abnormal	58 (18.7)
Mood	ICOPE	310	Abnormal	43 (13.9)
Memory	ICOPE	249	Abnormal	28 (11.2)
Vision	ICOPE	312	Abnormal	114 (36.5)
Hearing	ICOPE	308	Abnormal	39 (12.7)
≥1 abnormal domains	Composite	273	—	214 (78.4)

Abbreviations: MoCA, Montreal Cognitive Assessment; PHQ-2, Patient Health Questionnaire-2; GAD-2, Generalized Anxiety Disorder-2; EASI, Elder Abuse Suspicion Index; ADL, Activities of Daily Living; IADL, Instrumental Activities of Daily Living; ICOPE, Integrated Care for Older People.

**Table 3:** Age-Stratified Prevalence of Aging-Related Impairments.

Domain	N-Screened	Category	50–59, n (%)	60–64, n (%)	≥65, n (%)	*p*-Value
(MOCA) Cognitive impairment	103	≥26 (normal)	20 (55.6)	11 (47.8)	22 (50.0)	0.54 **
		18–25 (mild)	15 (41.7)	10 (43.5)	16 (36.4)	
		10–17 (moderate)	1 (2.8)	2 (8.7)	6 (13.6)	
(PHQ4) Depression	152		4 (9.1)	0 (0.0)	3 (4.1)	0.16 **
(GAD) Anxiety	190	GAD-2 ≥ 3	6 (10.3)	2 (4.7)	10 (11.2)	0.46
(EASI) Safety	190	≥1 positive	3 (5.2)	1 (2.3)	6 (6.7)	0.57 **
(Edmonton) Frailty	187	0–5 not frail	52 (89.7)	42 (97.7)	79 (91.9)	0.62 **
		6–7 vulnerable	4 (6.9)	1 (2.3)	5 (5.8)	
		8–9 mild frailty	1 (1.7)	0 (0.0)	2 (2.3)	
		10–11 moderate frailty	1 (1.7)	0 (0.0)	0 (0.0)	
(Katz) ADL	189	≥1 ADL limitation	6 (10.5)	7 (16.3)	6 (6.7)	0.23
(Lawton–Brody) IADL	190	≥1 IADL limitation	1 (1.7)	1 (11.1)	7 (7.9)	0.16 **
(ICOPE) ≥ 1 abnormal domains (%)	273		74 (77.9)	54 (81.8)	86 (76.8)	0.79

Data are presented as n (%). ** Low cell counts; results should be interpreted with caution. Abbreviations: MoCA, Montreal Cognitive Assessment; PHQ-2, Patient Health Questionnaire-2; GAD-2, Generalized Anxiety Disorder-2; EASI, Elder Abuse Suspicion Index; ADL, Activities of Daily Living; IADL, Instrumental Activities of Daily Living; ICOPE, Integrated Care for Older People.

**Table 4:** Prevalence of Aging-Related Impairments Stratified by Years Since Diagnosis.

Domain	N-Screened	Category	0–9 Years, n = 21 (10.82%)	10–19 Years, n = 44 (22.68%)	≥20 Years, n = 129 (66.49%)	*p*-Value
(MOCA) Cognitive impairment	103	≥26 (normal)	8 (61.5)	8 (34.8)	37 (55.2)	0.2279
		18–25 (mild)	4 (30.8)	14 (60.9)	23 (34.3)	
		10–17 (moderate)	1 (7.7)	1 (4.4)	7 (10.5)	
(PHQ-2) Depression	152		1 (7.7)	2 (5.4)	4 (3.9)	0.5504
(GAD) Anxiety	190	GAD-2 ≥ 3	2 (10.0)	2 (4.6)	14 (11.1)	0.4697
(EASI) Safety	190	≥1 positive	4 (20.0)	1 (2.3)	5 (4.0)	0.0231
(Edmonton) Frailty	187	0–5 not frail	18 (90.0)	41 (93.2)	114 (92.7)	0.7031
		6–7 vulnerable	2 (10.0)	3 (6.8)	5 (4.1)	
		8–9 mild frailty	0 (0.0)	0 (0.0)	3 (2.4)	
		10–11 moderate frailty	0 (0.0)	0 (0.0)	1 (0.8)	
(Katz) ADL	189	≥1 ADL/IADL limitation	2 (10.5)	3 (6.8)	14 (11.1)	0.7460
(Lawton–Brody) IADL	190	≥1 IADL limitation	2 (10.0)	0 (0.0)	7 (5.6)	0.1212
(ICOPE) ≥ 1 abnormal domains (%)	273		27 (77.1)	53 (81.5)	134 (77.5)	0.7782

Data are presented as n (%). Fisher’s exact test was used to confirm findings. Abbreviations: MoCA, Montreal Cognitive Assessment; PHQ-2, Patient Health Questionnaire-2; GAD-2, Generalized Anxiety Disorder-2; EASI, Elder Abuse Suspicion Index; ADL, Activities of Daily Living; IADL, Instrumental Activities of Daily Living; ICOPE, Integrated Care for Older People.

**Table 5:** Referrals, Services, and Clinical Interventions Following Screening.

Screening Domain	Referral or Service/Service Type	Participants Receiving Referral or Service, %
Cognitive impairment (MoCA)	Neurology/Geriatrics	3.2%
Depression (PHQ-2)/Anxiety (GAD-2)	Psychiatry/Behavioral Health	42.3%
Social risk/safety (EASI)	Social Work	42.9%
Functional limitation (Katz ADL/Lawton IADL)	PT/OT	8.8%
Frailty (Edmonton Frail Scale)	Exercise/Rehab	1.9%
Cardiometabolic risk (clinical + pharmacy assessment)	Pharmacy review	56.8%
Nutrition (ICOPE domain)	Nutrition counseling	12.6%
Intrinsic capacity impairment (ICOPE/multimorbidity)	Medical subspecialty referral	30.3%
Functional/social complexity (multidomain impairment)	Clinical care coordination	7.3%
Social risk (ICOPE/SDOH)	Food Insecurity	2.5%
Psychosocial/social well-being (ICOPE domain)	Peer Support	1.0%

Percentages reflect the overall HAP cohort and categories were not restricted to participants completing any single screening domain. Abbreviations: PT, physical therapy; OT, occupational therapy; SDOH, social determinants of health.

**Table 6:** Cardiometabolic Parameters at Baseline and Follow-Up.

Parameter	n Assessed at Baseline	Baseline (Mean ± SD or Median [IQR])	n Assessed at 6-Month Follow-Up	6 Month (±3) Follow-Up	n Used to Assess 6 Month Change	6 Month Change	n Assessed at 12 Month Follow-Up	12 Month Follow-Up	n Used to Assess 12 Month Change	12 Month Change
ASCVD risk score (%)	108	11.75 (6.05 −19.20)	74	9.90 (6.20–18.6)	67	−0.2 (−2.60–1.80)				
LDL cholesterol (mg/dL)	313	98 (75–123)	174	93.5 (74–119.8)	174	−4.5 (−1–3.3)	100	90.5 (73.8–105)	99	−7 (−1–18)
Systolic BP (mmHg)	317	133 (123–142)	247	132 (121.5–143)	247	−1 (−1.5–1)	130	132.5 (121.3–145)	130	−0.5 (−1.8–3)
Diastolic BP (mmHg)	317	80 (76–84)	247	80 (75–84)	247	0 (−1–0)	130	80 (74–85)	130	0 (−2–1)
BMI (kg/m^2^)	317	27.9 (24.9–31.9)	245	27.9 (25.1–31.1)	245	0 (0.1–0.8)	132	27.3 (22.8–30.3)	132	−0.7 (−2.1–1.6)
HbA1c (%), if available	314	5.7 (5.4–6)	167	5.7 (5.4–6.1)	166	0 (0–0.1)	98	5.7 (5.4–6)	98	0 (0–0)

Data are presented as median (interquartile range [IQR]). Abbreviations: ASCVD, atherosclerotic cardiovascular disease; LDL, low-density lipoprotein; BP, blood pressure; BMI, body mass index; HbA1c, hemoglobin A1c.

## Data Availability

Data supporting the findings of this study are available from the corresponding author upon reasonable request. Due to the inclusion of protected clinical information, the data are not publicly available.
